# Detection of SARS-CoV-2 infection by microRNA profiling of the upper respiratory tract

**DOI:** 10.1371/journal.pone.0265670

**Published:** 2022-04-05

**Authors:** Ryan J. Farr, Christina L. Rootes, John Stenos, Chwan Hong Foo, Christopher Cowled, Cameron R. Stewart

**Affiliations:** 1 CSIRO Health & Biosecurity, Australian Centre for Disease Preparedness, Geelong, Victoria, Australia; 2 Australian Rickettsial Reference Laboratory, University Hospital Geelong, Geelong Victoria, Australia; 3 Exios Bio LLC, Conshohocken, Pennsylvania, United States of America; All India Institute of Medical Sciences, INDIA

## Abstract

Host biomarkers are increasingly being considered as tools for improved COVID-19 detection and prognosis. We recently profiled circulating host-encoded microRNA (miRNAs) during SARS-CoV-2 infection, revealing a signature that classified COVID-19 cases with 99.9% accuracy. Here we sought to develop a signature suited for clinical application by analyzing specimens collected using minimally invasive procedures. Eight miRNAs displayed altered expression in anterior nasal tissues from COVID-19 patients, with miR-142-3p, a negative regulator of interleukin-6 (IL-6) production, the most strongly upregulated. Supervised machine learning analysis revealed that a three-miRNA signature (miR-30c-2-3p, miR-628-3p and miR-93-5p) independently classifies COVID-19 cases with 100% accuracy. This study further defines the host miRNA response to SARS-CoV-2 infection and identifies candidate biomarkers for improved COVID-19 detection.

## Introduction

Host responses to SARS-CoV-2 infection are currently being examined as biomarkers for both improved detection of pre- or asymptomatic COVID-19 cases [1] and the prognosis of COVID-19 severity [2–4]. In contrast to viral RNA molecules, host biomarkers such as microRNAs (miRNAs) are relatively abundant in the patient during the early pre-symptomatic period. MicroRNAs are small non-coding RNAs that regulate biological processes, including the host antiviral immune response. During the earliest phase of viral infection, prior to symptom onset and detectable virions, the pathogen triggers signaling cascades in the innate effectors of the host immune system. These first line responders (e.g. myeloids) react rapidly, releasing expressed miRNAs in circulation.

We recently characterised changes in the circulating miRNA profile of human plasma observed during SARS-CoV-2 infection [1]. With many molecular COVID-19 tests employing nasal or nasopharyngeal swabs as analytes, here we analysed the miRNA profile in nasal swabs derived from COVID-19 patients and uninfected controls.

## Materials and methods

### Ethics statement

The analysis of miRNAs from patient samples was approved by the CSIRO Human Research Ethics Committee (proposal # 2020_19). Formal consent was not obtained from patients due to anonymity. All patient information was de-identified and samples randomised prior to RNA isolation.

### Patient cohort information, sample collection and storage

Swabs of the anterior nares were collected by Barwon Health (Geelong, Australia) from members of the public undergoing COVID-19 testing between July and August 2020 (Table 1). Samples were also collected from persons defined as uninfected controls, who displayed no COVID-19 symptoms and returned negative SARS-CoV-2 PCR test results. Samples were collected by inserting swabs into patient nostrils (no more than 1.5 cm), then slowly rotated for a total of 15 sec, collecting as much nasal discharge as possible. Swabs were collected in universal transport medium and stored at -80°C until processed.

**Table 1:** Overview of patient information.

Patient	Collection date	Test result	SARS-CoV-2 CT Replicate 1	SARS-CoV-2 CT Replicate 2
NS001	6/08/2020	SARS-CoV-2 Positive	28.26	28.3
NS002	9/08/2020	SARS-CoV-2 Positive	29.11	29.51
NS004	9/08/2020	SARS-CoV-2 Positive	29.21	29.18
NS005	8/08/2020	SARS-CoV-2 Positive	20.95	20.9
NS007	6/08/2020	SARS-CoV-2 Positive	29.29	28.28
NS008	5/08/2020	SARS-CoV-2 Positive	35.05	37.56
NS009	9/08/2020	SARS-CoV-2 Positive	28.91	28.15
NS010	6/08/2020	SARS-CoV-2 Positive	21.19	19.53
NS011	8/08/2020	SARS-CoV-2 Positive	22.26	24.57
NS012	5/08/2020	SARS-CoV-2 Positive	34.46	35.63
NS013	7/08/2020	SARS-CoV-2 Positive	27.68	28.52
NS014	5/08/2020	SARS-CoV-2 Positive	21.57	23.24
NS015	19/07/2020	Negative	-	-
NS016	19/07/2020	Negative	-	-
NS017	19/07/2020	Negative	-	-
NS018	19/07/2020	Negative	-	-
NS019	19/07/2020	Negative	-	-
NS020	19/07/2020	Negative	-	-
NS021	19/07/2020	Negative	-	-
NS023	19/07/2020	Negative	-	-

### RNA isolation and next-generation sequencing (NGS)

Total RNA was isolated from 200 μL of sample using the miRNeasy micro kit (Qiagen) as per the manufacturer’s instructions with one modification: following lysis with Qiazol, glycogen (10 μg, Sigma Aldrich, G1767) was added as a carrier to each sample. Complementary DNA libraries were prepared using the QIAseq miRNA Library Kit with QIAseq miRNA NGS 48 Index IL (Qiagen) as per the manufacturer’s protocol (HB-2157-007 March 2020), with the following modifications: 5 μL of RNA was used as the template and the library amplification increased to 24 cycles. Libraries were analysed using the High Sensitivity DNA chip (Agilent) on the Agilent Bioanalyser 2100 to ensure correct insert size and minimal adapter or primer carryover. Libraries were sent to the Australian Genome Research Facility (AGRF) for 100 bp single end sequencing on the NovaSeq 6000 (Illumina).

### Data pre-processing and differential expression

Reads were trimmed of adapters to a read length of 18–26 nucleotides using CutAdapt. The remaining reads were reviewed using FastQC (www.bioinformatics.babraham.ac.uk/projects/fastqc/) to ensure high-quality data. miRNA identification and quantification were carried out using miRDeep2 against the most recent miRBase human reference (version 22). Read counts were normalised and differential expression analysis was performed in R using the DESeq2 package. An adjusted False Discovery Rate (FDR) of a p-value of <0.05 was used to identify differentially expressed miRNAs.

### Machine learning

All machine learning analysis was conducted using the scikit-learn [5] module in python. miRNA counts were scaled using either a standard z-score transformation or a robust scaler (where the median is removed and the data is scaled according to the interquartile range). Feature selection was performed using recursive feature elimination (RFE) to identify the miRNAs that contributed the most to the classification model. For binary classification, a logistic regression model was used. For multiclass classification, a linear support vector classifier was used. Once the optimal number of features (miRNAs) was selected, the data was PCA transformed. Each model underwent hyperparameter tuning using GridSearchCV. To assess the performance of the classification model, the data was randomly split into 70% labelled training data and 30% unlabelled test data, and the predicted classes of the test data samples were compared to the true classes. This process was repeated 1,000 times to ensure confidence in the classification performance. The machine learning models were assessed on their accuracy (how many of the predictions were correct), precision (how many of the predicted positives were true positives), and recall (how many of the true positives were found by the model). The logistic regression model was also assessed using the receiver operating characteristic area under the curve (ROC AUC), which is a succinct metric to describe a binary classification model [6].

### Statistics

Statistical analyses were performed using the SciPy v1.6.0 analysis package. All measurements were obtained from individual samples. Differences in qRT-PCR results were assessed using a one-sided Mann-Whitney U test due to the non-parametric nature of the fold-over-detectable transformation. Normality was tested using a combination skew and kurtosis test (scipy.stats.normaltest). A p-value <0.05 was considered significant.

## Results and discussion

**Fig 1:**
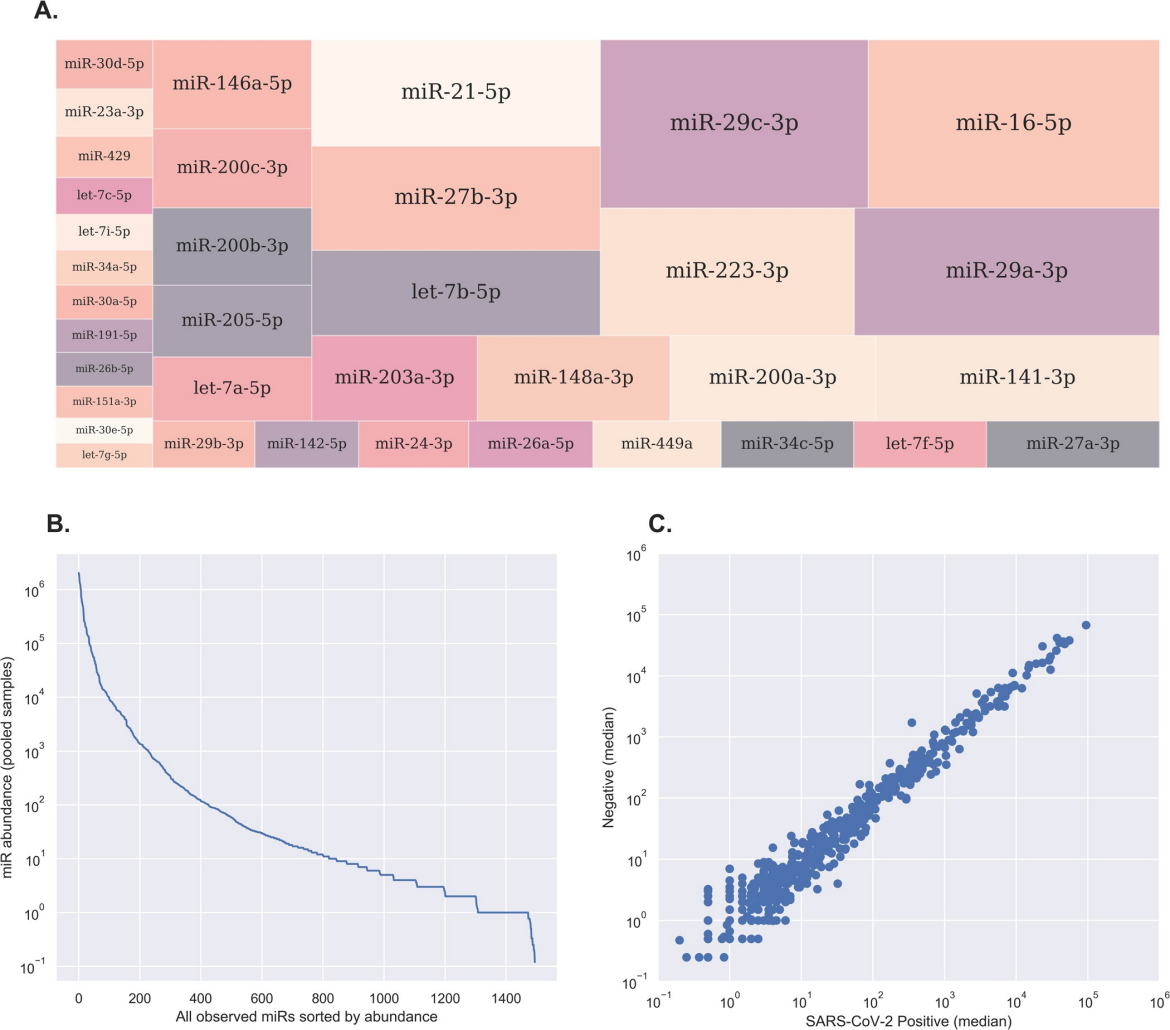
Overview of host-encoded miRNAs in nasal swab samples. **A,** Treemap plot displaying the relative abundance of the most highly-expressed miRNAs in nasal swab samples. The most prevalent miRNA was miR-16-5p, followed by miR-29a-3p, miR-29c-3p and miR-223-3p. **B**, Line plot showing the relative abundance of all host miRNAs identified in nasal swab samples. A total of 1495 miRNAs were detected across all 20 samples, of which 452 were detected at greater than 100 reads. **C**, Scatter plot illustrating inter-sample variance at the single-miRNA level. Each individual point represents a single mature miRNA, shown as the median of DESeq2-normalized read counts in each group and drawn in log_10_ scale on both axes. MiRNAs that lie exactly on the diagonal midline have equal expression in both groups, while miRNAs located further away from the midline are potential candidates for differential expression.

Small RNA seq resulted in 13–46 million (average 23 million) raw reads per sample, which have been submitted to the NCBI short read archive (SRA, project accession number PRJNA816999). Reads were trimmed of adaptors and filtered on length (18–26 nt) and quality, resulting in 2.4–10.6 million (average 5.4 million) reads per sample for further analysis. MiRDeep2 mapper was used to identify all known miRNA transcripts amongst the 20 samples (by mapping to the miRBase v22 human dataset) and read counts were determined for each mature miRNA transcript using miRDeep2 quantifier. A total of 1,495 different 5p or 3p mature miRNA transcripts were detected, corresponding to 1,097 different precursors. The most abundant miRNA in the nasal swab dataset was hsa-miR-16-5p, followed by hsa-miR-29c-3p, hsa-miR-29a-3p and hsa-miR-223-3p (Fig 1A). A total of 452 (28% of all detected miRNAs) had at least 100 reads (Fig 1B). Following data normalization, pairwise analyses was performed at the single-miRNA level using median normalised read counts from infected vs uninfected samples, revealing a high degree of similarity (Fig 1C). By applying a stringent data filtering and normalisation strategy, miRNA expression between different individuals was demonstrated to exhibit a low level of biological and technical variation, confirming the suitability of this dataset to assess changes in miRNA expression between patient groups.

**Fig 2:**
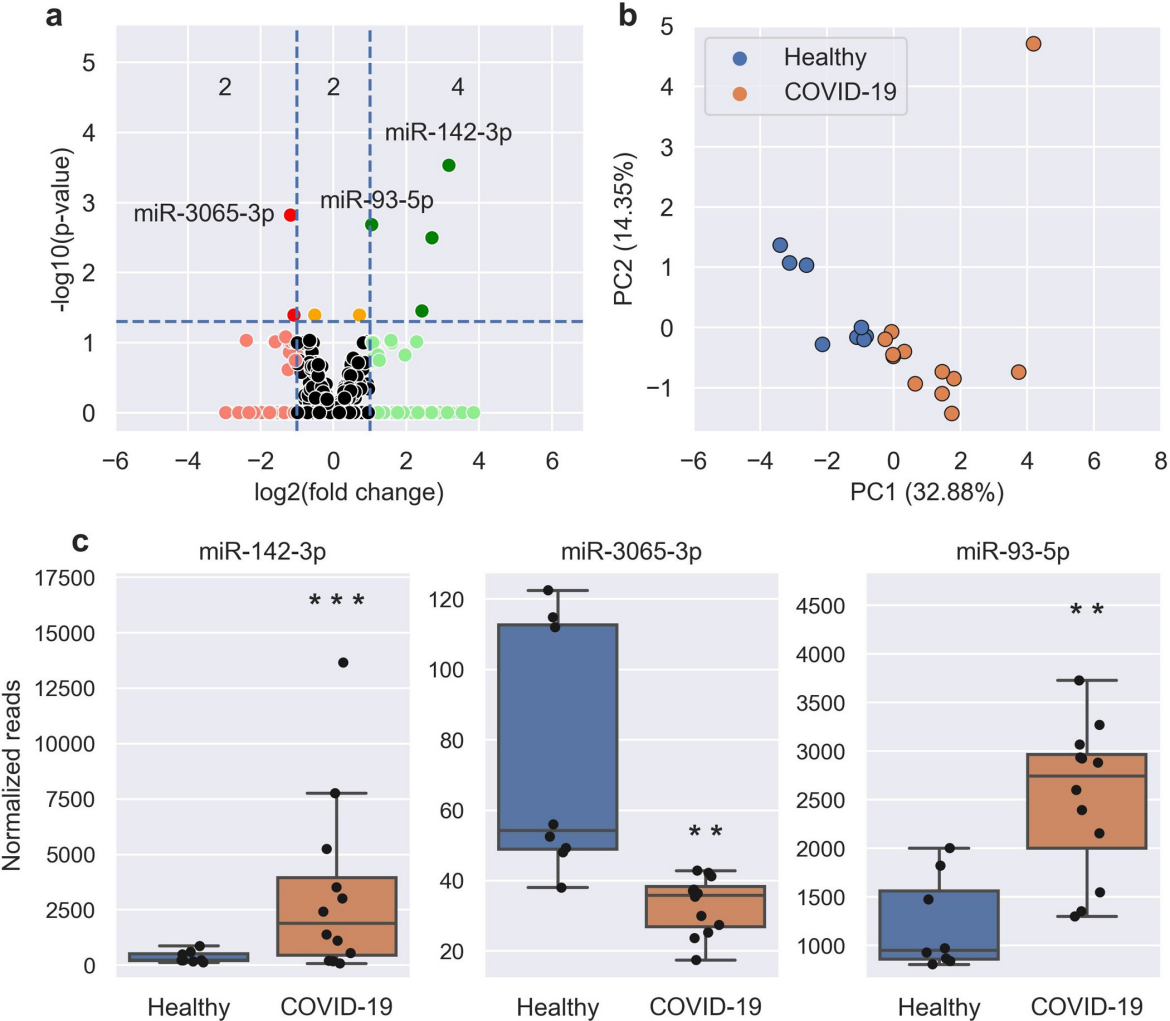
SARS-CoV-2 induces significant chances in the miRNA profile from patient nasal swabs. **A,** Volcano plot showing the increased (green) and decreased (red) DE miRNAs in COVID-19 patients when compared to healthy controls. Horizontal dotted line is the p-value cut-off (False Discovery Rate, FDR<0.05) and the vertical lines are the fold change cut-off (>2 FC). Orange miRNAs are statistically significant but are not >2 FC. The number of statistically significant miRNAs (adjusted P-value <0.05) in each section are shown: <-1 Log2 FC (2 miRNAs), between –1 and 1 Log2 FC (2 miRNAs), and >1 Log2 FC (4 miRNAs). The most up-regulated, down-regulated, and statistically significant miRNAs have been labelled. **B,** PCA plot showing the separation of healthy (blue) and COVID-19 (orange) samples using the 8 DE miRNAs. **C,** Boxplots of select miRNAs in healthy (blue) and COVID-19 (orange) samples. Boxes are the 25^th^ - 75^th^ percentile, line is the median, and whiskers are 1.5x IQR. ** p-value < 0.01, *** p-value < 0.001.

**Fig 3:**
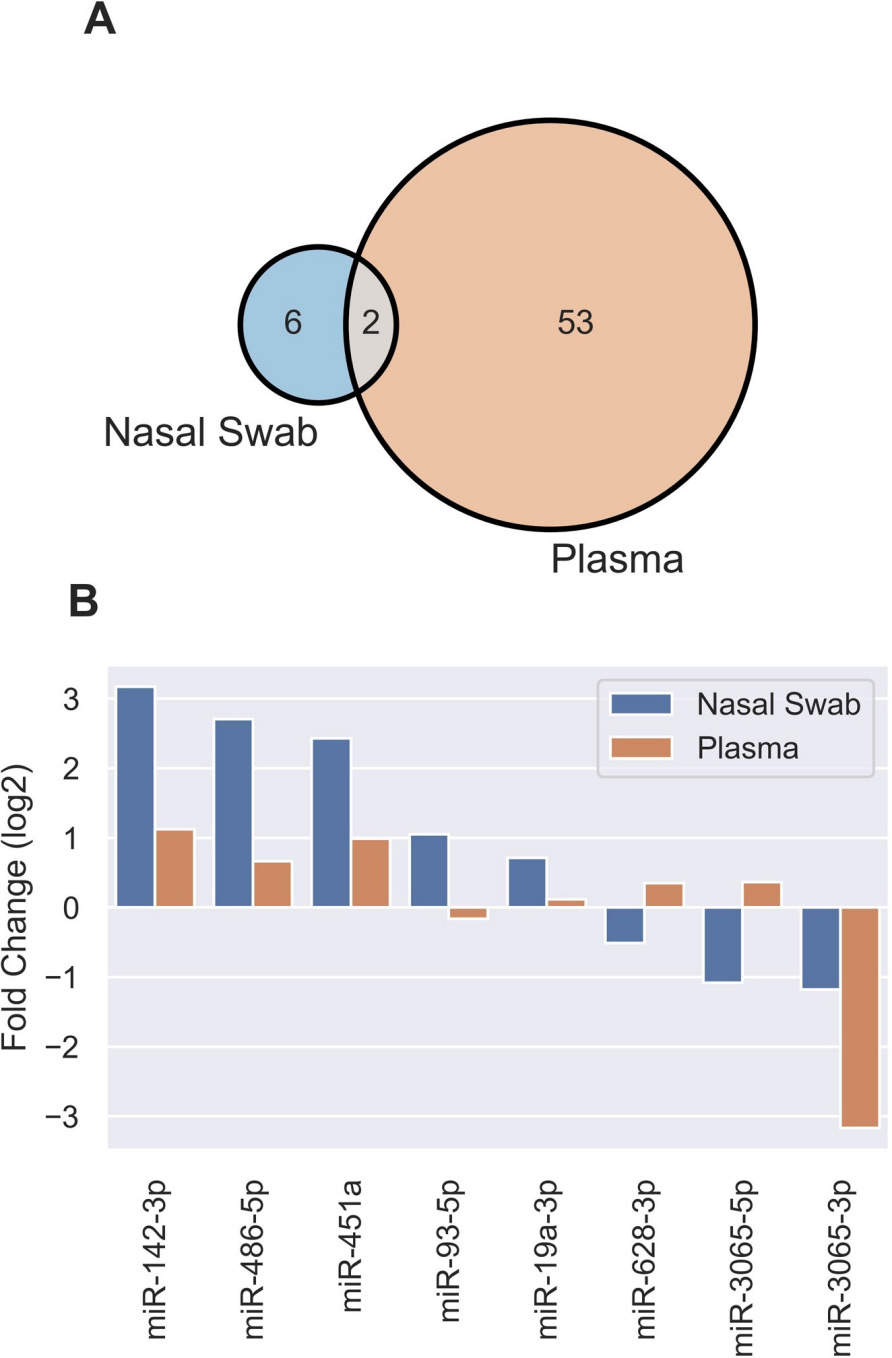
Comparisons of DE miRNAs induced by SARS-CoV-2 infection in nasal swabs and plasma. **A,** Venn diagram identifying DE miRNAs in plasma and nasal swabs miRNA datasets. **B,** Results from DE miRNA analysis from nasal swab and plasma datasets for the 8 miRNAs listed.

Using DESeq2 to perform count-based differential expression (DE) testing, a subset of miRNAs that were up- or down-regulated in COVID-19 cases relative to uninfected controls were identified (Fig 2A, S1 Table). Using a False Discovery Rate (FDR) adjusted p-value <0.05, log_2_ fold change (FC) >1 and baseMean >5, this dataset consisted of 6 miRNAs, of which four were up-regulated (elevated in infected patients) and two were down-regulated. An additional two miRNAs were significantly DE in COVID-19 patients with log_2_FC values <1. The most highly up-regulated candidates in COVID-19 patients were miR-142-3p (Fig 2A), miR-486-5p, and miR-451a, while the most down-regulated were miR-3065-3p (Fig 2A) and miR-3065-5p. The most statistically significant change was seen in miR-142-3p. Unsupervised analysis of variance using principal components analysis (PCA) involving the eight DE miRNAs showed tight clustering of patient groups (Fig 2B). Differences in miRNA expression for miR-142-3p, miR-3065-3p and miR-93-5p are shown in Fig 2C. Upon comparing miRNAs differentially expressed in COVID-19 patients in nasal swabs and plasma [1], two miRNAs (miR-142-3p and miR-3065-3p) were DE in both datasets (Fig 3A), while miRNAs DE in nasal swabs for the most part showed agreement in terms of upregulation or downregulation without being statistically significant in plasma (Fig 3B).

**Fig 4:**
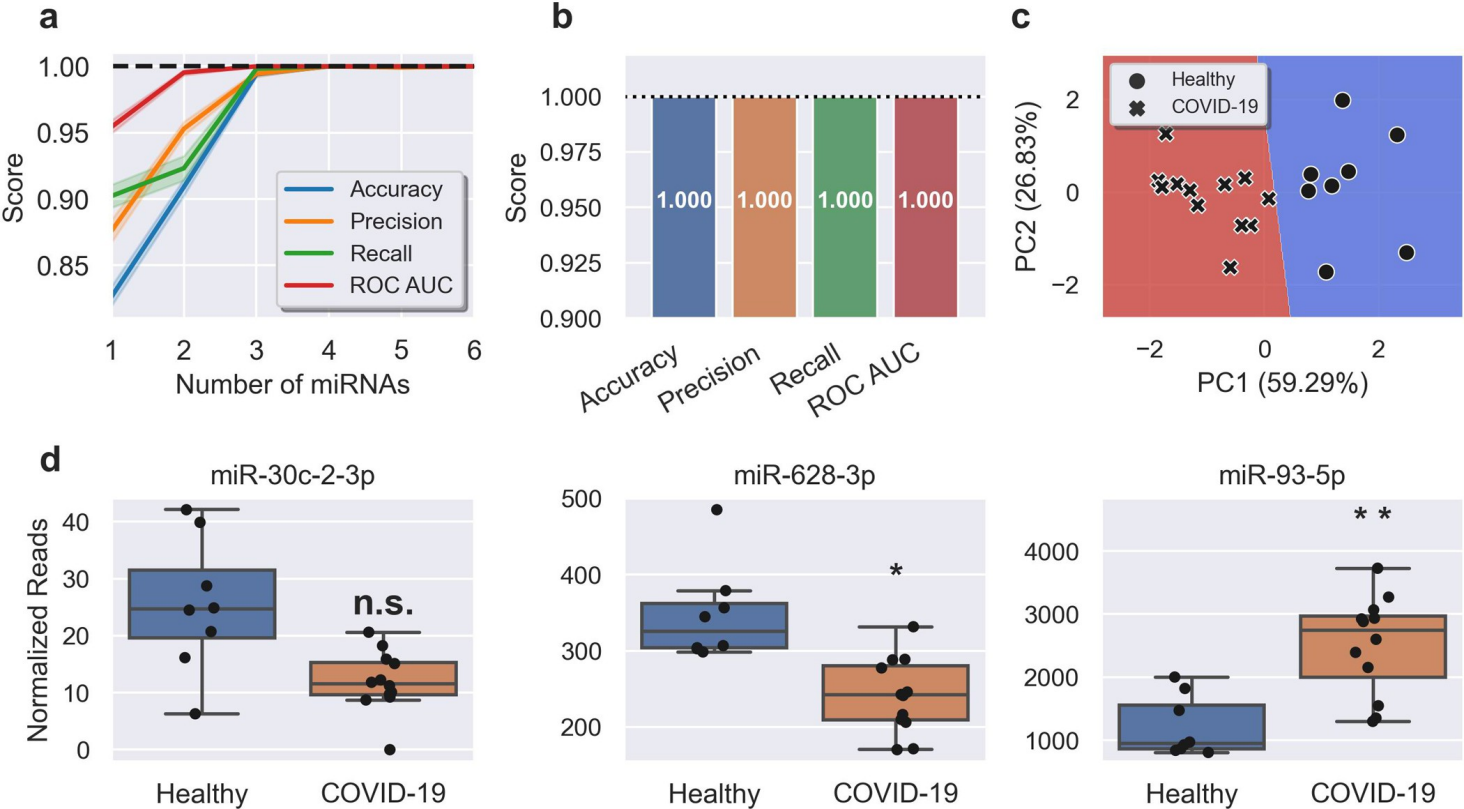
A miRNA signature in nasal swabs classifies COVID with 100% accuracy. **A,** Feature (miRNA) selection lineplot showing the impact of increasing numbers of miRNAs on the performance of a logistic regression model. MicroRNAs were selected using recursive feature elimination to identify the most important miRNAs. Each combination of miRNAs was randomly assessed 1,000 times. Shaded areas are the 95% CI, and the dotted line is a perfect (100%) score. **B,** Barplot showing the average score of the three-miRNA signature in predicting healthy controls and COVID-19 patients. Error bars are the 95% CI after 1,000 random iterative assessments. **C,** Decision boundary graph showing the logistic regression decision point (solid black line) and the probability a person is infected with SARS-CoV-2 (blue to red shading). Datapoints are healthy (circles) and COVID-19 (crosses) samples. **D,** Boxplots of each of the signature miRNAs in healthy (blue) and COVID-19 (orange) samples. Boxes are the 25^th^ - 75^th^ percentile, line is the median, and whiskers are 1.5x IQR. * FDR adjusted p-value < 0.05, ** FDR adjusted p-value < 0.01. n.s. non-significant.

We next investigated if, similar to miRNA profiles in plasma, changes in the nasal swab profile could independently classify SARS-CoV-2 infection. A supervised machine learning method was implemented for the identification of the most predictive miRNAs and refined to identify the minimum number needed for accurate prediction. The most predictive miRNAs were selected using recursive feature elimination (Fig 4A). Measuring three miRNA targets (miR-30c-2-3p, miR-628-3p and miR-93-5p) in combination gave a model with 100% accuracy, 100% precision and 100% recall, with a ROC AUC of 1.0 (Fig 4B). This composite biomarker was comprised of two miRNAs DE in COVID-19 patients (miR-628-3p (downregulated) and miR-93-5p (upregulated)) and miR-30c-2-3p, which was not DE (Fig 4D). A decision boundary graph showed clear distinctions between healthy and infected patients based on these three miRNAs (Fig 4C).

Several miRNAs DE in COVID-19 patient nasal swabs are associated with inflammation. Elevated expression of miR-142-3p has been reported in Crohn’s disease and ulcerative colitis, where elevated levels of miR-142-3p are observed in colon, blood and saliva [7]. Separate studies demonstrated a correlation between elevated miR-142-3p and circulating IL-6 levels in inflammatory bowel disease [8] and miR-142-3p/IL-6 production in dendritic cells stimulated with lipopolysaccharide (LPS) [9]. Studies using luciferase reporters carrying wild-type and altered IL-6 3’UTR confirm *IL6* as a miR-142-3p target gene [9], while miR-142-3p acts in a dose-dependent manner to inhibits *IL6* transcription in polymorphonuclear leukocytes stimulated with LPS [10]. miR-93-5p, also up-regulated in COVID-19 anterior nasal tissues, inhibits the production of IL-6, TNF and IL-1β in osteoarthritis and diabetic nephropathy models through regulation of high mobility group proteins HMGB1 and HMGA2, respectively [11, 12]. IL-6 is one of the key mediators of viral cytokine storm and inflammation in patients with severe COVID-19 [13]. It is intriguing to speculate that miR-142-3p and miR-93-5p are induced to counteract potentially deleterious effects of elevated IL-6 in COVID-19 patients, a response associated with respiratory failure and death [14]. Intriguingly, the most downregulated miRNA in nasal swabs from COVID-19 patients, the relatively poorly-characterised miR-3065-3p, is also down-regulated in inflamed placental tissue and significantly reduced by LPS stimulation [15]. Additionally, other miRNAs responsive to SARS-CoV-2 infection has no known links to inflammation but have been observed in infection [16, 17].

Further studies are planned to address limitations in this study, particularly relating to the analytical specificity of miRNA profiles associated with COVID-19. This study has not investigated host miRNA responses to infections other than SARS-CoV-2, with other pathogens causing lower and upper respiratory tract infections of particular interest. While it is interesting to note that circulating miRNA profiles in animal models of COVID-19 and influenza are distinct [1], and miRNA responses to seasonal influenza viruses differ according to virus subtype both *in vivo* [18] and *in vitro* [19], further work is required to define the robustness and specificity of miRNA responses to particular pathogens. Such studies should also consider chronic diseases, with miR-142-3p for example associated with distinct inflammatory conditions. Furthermore, while our primary objective in this study was to discover unique miRNA profiles in COVID-19 cases, independent of their disease state, severity or chronology, future studies may investigate miRNA correlates of COVID-19 severity for prognostic indications.

## Conclusion

One of the more dangerous features of COVID-19 is its ability for sustaining human-to-human transmission pre- and asymptomatically [20]. U.S. CDC estimates that 40% of transmission occurs prior to symptom onset [20]. Furthermore, approximately 35% of COVID-19 infections remain asymptomatic throughout the entire course of the disease [16]. These traits of COVID-19 have facilitated its rapid spread leading to the current deadly global pandemic, and highlights that innovations are required to fill gaps in the SARS-CoV-2 diagnostic landscape. Here we have shown that positive COVID-19 PCR test results correspond to a change in the nasal swab miRNA profile that can independently classify disease cases. Further studies involving larger patient groups, including pre-symptomatic, asymptomatic and different (e.g. severity, variants) infections are planned to assess whether this pattern is observed during the COVID-19 incubation period (median 6.5 days) and would thus have real-world application for improved disease detection or prognosis. As miRNA responses are reflective of the host response to infection, miRNA biomarkers could also provide clinical utility in the provision of infection evidence to reduce false-negative rates with PCR testing [21].

## Supporting information

S1 Table DE miRNAs in COVID-19 cases. (XLSX)
